# Disruption of cholinergic neurotransmission, within a cognitive challenge paradigm, is indicative of Aβ-related cognitive impairment in preclinical Alzheimer’s disease after a 27-month delay interval

**DOI:** 10.1186/s13195-020-00599-1

**Published:** 2020-03-24

**Authors:** Jessica Alber, Paul Maruff, Cláudia Y. Santos, Brian R. Ott, Stephen P. Salloway, Don C. Yoo, Richard B. Noto, Louisa I. Thompson, Danielle Goldfarb, Edmund Arthur, Alex Song, Peter J. Snyder

**Affiliations:** 1 Department of Biological & Pharmaceutical Sciences, College of Pharmacy, University of Rhode Island, 75 Lower College Road, 2nd Floor, Kingston, RI USA; 2 Ryan Institute for Neuroscience, University of Rhode Island, Kingston, RI USA; 3 Department of Psychiatry and Human Behavior, Warren Alpert Medical School of Brown University, Providence, RI USA; 4 Cogstate Ltd., Melbourne, Victoria Australia; 5 The Florey Institute of Neuroscience and Mental Health, The University of Melbourne, Melbourne, Victoria Australia; 6 Department of Neurology, Warren Alpert Medical School of Brown University, Providence, RI USA; 7 Department of Radiology, Warren Alpert Medical School of Brown University, Providence, RI USA; 8 Banner Alzheimer’s Institute, Phoenix, AZ USA; 9 Brown University, Providence, RI USA

**Keywords:** Alzheimer disease, Preclinical Alzheimer’s disease, Early detection, Cholinergic, Cognition, Biomarkers, Early diagnosis, Anticholinergic drugs, Scopolamine, Beta-amyloid protein

## Abstract

**Background:**

Abnormal beta-amyloid (Aβ) is associated with deleterious changes in central cholinergic tone in the very early stages of Alzheimer’s disease (AD), which may be unmasked by a cholinergic antagonist (J Prev Alzheimers Dis 1:1–4, 2017). Previously, we established the scopolamine challenge test (SCT) as a “cognitive stress test” screening measure to identify individuals at risk for AD (Alzheimer’s & Dementia 10(2):262–7, 2014) (Neurobiol. Aging 36(10):2709-15, 2015). Here we aim to demonstrate the potential of the SCT as an indicator of cognitive change and neocortical amyloid aggregation after a 27-month follow-up interval.

**Methods:**

Older adults (*N* = 63, aged 55–75 years) with self-reported memory difficulties and first-degree family history of AD completed the SCT and PET amyloid imaging at baseline and were then seen for cognitive testing at 9, 18, and 27 months post-baseline. Repeat PET amyloid imaging was completed at the time of the 27-month exam.

**Results:**

Significant differences in both cognitive performance and in Aβ neocortical burden were observed between participants who either failed vs. passed the SCT at baseline, after a 27-month follow-up period.

**Conclusions:**

Cognitive response to the SCT (Alzheimer’s & Dementia 10(2):262–7, 2014) at baseline is related to cognitive change and PET amyloid imaging results, over the course of 27 months, in preclinical AD. The SCT may be a clinically useful screening tool to identify individuals who are more likely to both have positive evidence of amyloidosis on PET imaging and to show measurable cognitive decline over several years.

## Background

To best slow the neuropathologic cascade and deterioration of cognitive functions due to Alzheimer’s disease (AD), neuroprotective treatments will likely be most efficacious if administered during the preclinical stages of the disease [1, 2]. However, reliably identifying healthy individuals at high risk of developing AD currently requires biomarkers that are either invasive, expensive, and/or labor intensive to obtain [3–5]. Complex and expensive diagnostic procedures, such as imaging of neocortical amyloid and tau protein aggregation by positron emission tomography (PET), are limited to patients and facilities with access to the necessary resources and expertise [6, 7]. There remains a need for low-cost, minimally invasive screening tools for AD risk that may be readily administered by point-of-care providers [8].

We have previously proposed a cognitive stress test with sensitivity to detect the earliest neuropathologic changes in the basal forebrain cholinergic system that herald incipient AD [9]. The test involves the administration of a micro-dose of the muscarinic anticholinergic agent, scopolamine hydrobromide (0.2 mg, subcutaneous injection [s.c.]) prior to completion of a well-validated cognitive test previously shown to be sensitive to the manipulation of cholinergic tone [10], in order to unmask prodromal cognitive deficits indicative of cholinergic system changes and cortical beta-amyloid (Aβ) aggregation in older adults [1]. We explored the clinical utility of this scopolamine challenge test (SCT) in a longitudinal study of 63 mid-life adults (mean age = 62.79 years) with two known risk factors for AD. All participants completed the SCT at baseline, as well as amyloid PET imaging and neuropsychological assessments at baseline and at a 27-month follow-up exam. We have previously reported the baseline exam results from this study, which suggest that the use the SCT may be an effective way of identifying cognitively normal (CN) adults who demonstrate abnormal neocortical Aβ protein aggregation on PET imaging and are therefore likely in the preclinical stage of AD [11].

In this brief report, we present the 27-month follow-up results from the participants in this same study, with the aim of discerning whether the SCT may be a reliable indicator of continued risk for disease progression.

## Methods

### Participants

Sixty-three adults aged between 55 and 75 years, with a self-reported first-degree family history of AD and subjective memory complaints, were recruited from two memory disorder clinics in Rhode Island and broad advertising to the community. All participants underwent a detailed medical screening interview to exclude those who were diagnosed with MCI or AD, had a history of neurological or psychiatric disorder, were afflicted by any significant systemic illness or unstable medical condition, or used medications known to affect cognition. Inclusion criteria were a Mini-Mental State Examination (MMSE) total score > 27 and performance within normal limits on a battery of cognitive tests with demonstrated sensitivity to preclinical and prodromal AD (see Table 1). Participants were separated into high- (*N* = 15) versus low-Aβ (*N* = 48) groups on a post hoc basis, but the study team was kept blind to PET amyloid status until all baseline exams were completed and the results were reported by Lim et al. [11]. Table 1 summarizes the demographic and clinical characteristics of the study sample. The study was approved by and complied with the regulations of Rhode Island Hospital’s Institutional Review Board. All participants provided written informed consent.

**Table 1:** Demographic and clinical characteristics at baseline exam, for the full sample and for the two subgroups (those who either failed or passed the scopolamine challenge test (SCT) at their baseline exams)

	Main outcome	Full sample (*n* = 58)	SCT fail (*n* = 28)	SCT pass (*n* = 30)		
		*N* (%)	*N* (%)	*N* (%)	*p*	Cohen’s *d*
Sex	No. of female	38 (60%)	20 (71%)	18 (60%)	.296	–
*APOE*	No. of ε4 carriers	30 (48%)	17 (58%)	13 (43%)	.559	–
		Mean (SD)	Mean (SD)	Mean (SD)	*p*	
Age	No. of years	63.06 (5.42)	63.93 (6.31)	62.44 (5.04)	.349	0.28
Education	No. of years	17.21 (2.77)	17.47 (3.46)	17.14 (2.55)	.689	0.12
SUVr (neocortex)	Standardized uptake value ratio	**1.023 (0.19)**	**1.10 (0.24)**	**0.95 (0.09)**	**.000**	**2.47**
SUVr (anterior cingulate)	Standardized uptake value ratio	**1.064 (0.22)**	**1.197 (0.27)**	**0.984 (0.10)**	**.000**	**2.95**
GDS	Total score	1.86 (2.16)	1.60 (1.45)	1.94 (2.35)	.602	− 0.16
DASS Depression Subscale	Total Depression Subscale Score	3.56 (6.70)	2.60 (2.59)	3.87 (7.56)	.526	− 0.19
DASS Anxiety Subscale	Total Anxiety Subscale Score	2.73 (4.53)	2.40 (3.58)	2.83 (4.83)	.752	− 0.09
DASS Stress Subscale	Total Stress Subscale Score	6.73 (6.77)	6.67 (4.55)	6.74 (7.38)	.969	− 0.01
MAC-Q	Total score	21.80 (3.57)	21.35 (3.94)	21.28 (3.27)	.501	0.15
Body mass index	Body mass index	26.69 (5.50)	28.66 (7.95)	26.07 (4.41)	.113	0.48
MMSE	Total score	29.05 (1.02)	28.93 (1.16)	29.08 (0.99)	.624	− 0.15
GMLT Moves/Second (MPS)	Total correct moves/second	0.81 (0.20)	0.78 (0.20)	0.81 (0.19)	.573	− 0.17
GMLT Total Errors (TER)	Total no. of errors	55.60 (14.38)	53.21 (16.21)	52.79 (14.80)	.430	− 0.22
GMLT Composite	Standardized *z*-score	− 0.02 (0.81)	− 0.08 (0.89)	− 0.01 (0.79)	.764	− 0.09
ISLT Total Recall	Total words recalled (three learning trials)	25.62 (4.20)	24.04 (4.41)	27.85 (4.16)	.368	− 0.28
One Card Learning task	Accuracy of performance	1.01 (0.10)	1.05 (0.03)	1.10 (0.10)	.085	− 0.40
One Back task	Accuracy of performance	1.45 (0.11)	1.40 (0.14)	1.46 (0.10)	.063	− 0.54

*Note: *APOE* apolipoprotein, *SUVr* standardized uptake value ratio, *GDS* Geriatric Depression Scale, *DASS* Depression, Anxiety, and Stress Scale, *MAC-Q* Memory Complaints Questionnaire, *MMSE* Mini-Mental State Examination, *GMLT* Groton Maze Learning Task, *ISLT* International Shopping List Test; bolded values are significant at the *p* < .001 level

### Procedure

All participants completed the SCT at baseline, as described by Lim et al. [11]. Briefly, each subject completed a practice on the cognitive test (the Groton Maze Learning Test [GMLT]; www.cogstate.com), followed by a larger battery of cognitive tests (see Table 1) and a pre-dosing baseline GMLT test. Subjects then received an injection of scopolamine (0.2 mg s.c.). Following confirmation that vital signs had returned to pre-dose levels, the GMLT was re-administered at 1, 3, 5, 7, and 8 h post dosing. All subjects who failed to return to their own baseline performance level on the GMLT by five (5) hours post dosing were considered to have “failed” the SCT and thus were considered at increased risk for preclinical AD [11]. Participants also completed a cheek swab for apolipoprotein E (APOE) genotyping as an indicator of genetic AD risk at baseline, and florbetapir amyloid PET imaging at the baseline and 27-month exams, along with repeat cognitive assessments at 9, 18, and 27 months. Refer to the procedure as described by Lim et al. [11] for more details.

### Measures

All cognitive and psychological measures have been described by Lim et al. [11]. The neuropsychological tests assessed verbal memory (International Shopping List Test [ISLT; [12]), visual memory (Cogstate One Card Learning [OCL; [13] task), and working memory (Cogstate One Back [OBK; [13] task) (see Table 1). The Mini-Mental Status Exam (MMSE) was used to assess general cognitive function, and participants’ mood was measured using the 15-item Geriatric Depression Scale (GDS [14]) and the Depression, Anxiety and Stress Scale (DASS [15]). Subjective memory impairment was determined using the Memory Complaint Questionnaire (MAC-Q [16]). All measures were administered by trained staff supervised by a neuropsychologist.

As noted above, the GMLT was used in the SCT to detect/measure cholinergic-mediated disruptions in cognitive function following scopolamine administration. The GMLT, created by one of the authors (P.J.S.), is a computerized neuropsychological test of spatial working memory, learning efficiency, and error monitoring. The design and task requirements for the GMLT have been previously well described [10, 17]. Briefly, the task requires the participant to find a hidden pathway through a grid from the top left corner to a flag in the bottom right corner. The trial ends once the participant reaches the bottom right corner of the grid. Each participant completes five successive learning trials. The three main outcome measures of the GMLT are (1) mean correct moves per second across the five learning trials (MPS); (2) total number of errors made across the five learning trials (TER); and (3) total number of rule break errors made across the same five learning trials (RER). These three measures were standardized against the group baseline mean and standard deviation, and then averaged to form a GMLT composite.

### Aβ PET imaging

A 370 MBq (10 mCi +/− 10%) bolus injection of F-florbetapir [18] was administered intravenously at baseline and once again at 27 months. Approximately 50 min post-injection, a 20-min PET scan was performed with head CT scan for attenuation correction purposes. PET standardized uptake value (SUV) data were summed and normalized to the whole cerebellum SUV, resulting in a region-to-cerebellum ratio termed SUV ratio (SUVr). An SUVr threshold of ≥ 1.1 was used to discriminate between Aβ− and Aβ+; however, rather than using an average of recommended cortical areas [19], we defined Aβ+ as individuals with anterior cingulate (AC) SUVr ≥ 1.1. This definition was chosen for the following reasons: (1) we would not expect widespread neocortical amyloidosis in the very early preclinical stage of AD [20] in this sample, given the relatively young age of participants (i.e., on average 10 years younger than is typically the case for clinical trials cohorts [21, 22]), (2) the relationship between the AC and early changes in cholinergic tone [23, 24], and (3) emerging research suggests that increased Aβ burden, in the AC specifically, is highly related to memory changes in early AD [25, 26]. Table 1 also provides between-group differences on the established measure of total neocortical SUVr (averaged over six regions of interest [19]). For all cases, Aβ positivity was confirmed by consensus over-read by two board-certified radiologists who were also board-certified in Nuclear Medicine.

### Data analysis

All analyses were performed using JMP statistical software, version 12.0.1 (SAS Institute). The delayed free recall portion of the ISLT (a 12-item word-list learning test) was the key cognitive outcome measure for determining the clinical utility of the SCT to predict relative cognitive impairments at 27 months post-screening. This measure was chosen to avoid unnecessary and potentially misleading multiple comparisons, and because there is now clear evidence in the literature that verbal episodic memory is one of the earliest and most reliable domains to show AD-associated cognitive change [12, 27]. All subjects were separated into those who failed vs. passed the SCT at baseline (following [11]), and these groups were compared with two-way ANCOVA analyses (with age and APOE status entered as covariates) to determine (1) any main effects for ISLT delayed recall performance and (2) any amyloid PET imaging differences, at the 27-month outcome exam.

Next, a generalized estimating equation (GEE; [28]) was used to compare within-subject changes over the four exam time points, with respect to performance on both the ISLT and the GMLT between patients who passed and patients who failed the SCT. These differential changes were tested using a set of orthogonal linear contrasts: a 7-coefficient test for differences in linear trends across the four visit time points. GEEs are generalized linear models wherein the within-subject nesting is accounted for in the residual error of the model [28]. The selection of distribution for our models was guided based on the theory that simple counts ought to be Poisson distributed, and confirmed by model residuals. A compound symmetry variance-covariance structure was used with classical sandwich estimation to adjust for any misspecification [29]. To test the effect of SCT pass/fail on change in amyloid burden over time, we computed the absolute difference between baseline ACC SUVr and the 27-month ACC SUVr. We conducted an independent samples *t*-test for the SCT pass vs. fail group, with the absolute difference in the ACC SUVr (change in amyloid burden) as the dependent variable. Cohen’s *d* was used as the effect size measure. To determine whether SCT fail/Aβ PET+ subjects decline in episodic memory (measured using ISLT delayed recall score) compared to SCT fail/Aβ− subjects, we conducted a mixed model ANCOVA, co-varying for age. *P* values < 0.05 were considered statistically significant for all analyses.

### Receiver operating characteristic (ROC) analysis of SCT for amyloid PET status at baseline

The SCT was only administered at baseline and not at the 27-month follow-up visit. However, since the amyloid burden and absolute change in amyloid burden (ACC SUVr and absolute change in ACC SUVr) were consistently higher in the SCT fail group than the SCT pass group at both baseline and 27 months, we assumed that an ROC analysis of SCT for Aβ PET status at baseline would be similar to that at 27 months follow-up. Therefore, to test the sensitivity and specificity of SCT for amyloid PET status, we first found the proportion of Aβ+ participants in the SCT fail/pass group at baseline using a chi-square test with Yate’s correction. Next, an ROC analysis was conducted, examining SCT sensitivity and specificity for detection of amyloid burden on Aβ PET. The highest Youden index was used to set our cutoff and the corresponding sensitivity and specificity values. While SCT outcomes were dichotomized for other analyses, continuous GMLT composite scores [11] during the SCT were used for the ROC analysis.

## Results

In total, 58 of 63 participants completed all four exams over the 27-month study period (92% retention rate). Four subjects were lost to follow-up either because of relocation of their homes or loss of interest. One subject was diagnosed with progressive supranuclear palsy after the 18-month visit and was excluded from the study. The amount of neocortical Aβ aggregation and APOE genotype were unknown at the time of assessment and were not used to determine enrollment. Although Aβ status and APOE genotyping were conducted as a part of the study protocol, researchers remained blinded to these results throughout testing.

Of the 58 participants that completed all exam visits, there were 38 females, 11 of whom were Aβ+ on PET imaging. Four males were Aβ+ on PET imaging. There were no significant differences between SCT fail and SCT pass groups with respect to sex (*p* = 0.296). Likewise, there were no group differences in the proportion of individuals with the APOE ε4 genetic risk marker for AD. The mean age for the total sample was 63 years old and this sample had an average of 17 years of education. All relevant demographic information, for the entire sample, and broken down by SCT pass and SCT fail groups, is provided in Table 1. There were no group differences with respect to body mass index, subjective memory complaints, and cognitive performance at baseline exam (on any of the tests described above). By definition, both groups significantly differed with respect to neocortical amyloid aggregation as measured via PET imaging (*p* < .001).

With respect to genetic risk for AD, 17 of 28 individuals (58%) in the SCT fail group (those who failed the SCT at the time of their baseline exams) had at least one copy of the APOE ε4 allele, whereas 13 of 30 individuals (43%) in the SCT pass group (those who passed the SCT at the time of their baseline exams) had at least one copy of the APOE ε4 allele. Hence, more subjects in the SCT fail group presented with this additional risk factor for AD, but due to small sample sizes, we were not able to further evaluate the specific effect of APOE genetic risk on the relationship between SCT performance at baseline and cognitive performance 27 months later. We did, however, co-vary for APOE status in the analyses described below.

### Relationship between pass/fail on SCT at baseline and florbetapir PET Aβ binding 27 months later

Consistent with our initial findings at the time of the baseline PET scan reported previously [11], at 27 months follow-up, subjects who failed the SCT (*n* = 28) continued to show significantly greater amyloid binding in the ACC (mean SUVr = 1.197) compared to those who passed the SCT (*n* = 30) at baseline (mean SUVr = 0.984) after controlling for main effects of age (df = 1, MS = 24.87, F = 8.734, *p* = 0.0046), with a moderate-to-large effect size difference between groups (Cohen’s *d* = 0.75). There was no significant difference between the SCT pass and SCT fail groups in age (*p* = .81). The SCT fail group (0.106 ± 0.086) had significantly greater amyloid accumulation over 27 months in the ACC (measured using mean change in Aβ PET SUVr between baseline and 27 month follow-up) than the SCT pass group (0.064 ± 0.055), *t*(54) = 2.21, *p* = 0.031, with a moderate effect size (Cohen’s *d* = 0.58).

### Relationship between pass/fail on SCT at baseline and episodic verbal memory performance 27 months later

After controlling for age and APOE status, no group differences were found in performance on our chosen measure of episodic memory (ISLT delayed recall), at the time of the initial baseline exam, between those who failed (*n* = 31) versus passed (*n* = 32) the SCT (*p* = 0.93). However, at the time of the last study visit 27 months later, those who failed the SCT at the baseline exam performed significantly worse on the ISLT delayed recall test than those who passed the SCT at baseline (df = 1, MS = 1799.95, F = 5.337, *p* = 0.025), with a moderate effect size difference between groups (Cohen’s *d* = 0.58).

**Fig. 1:**
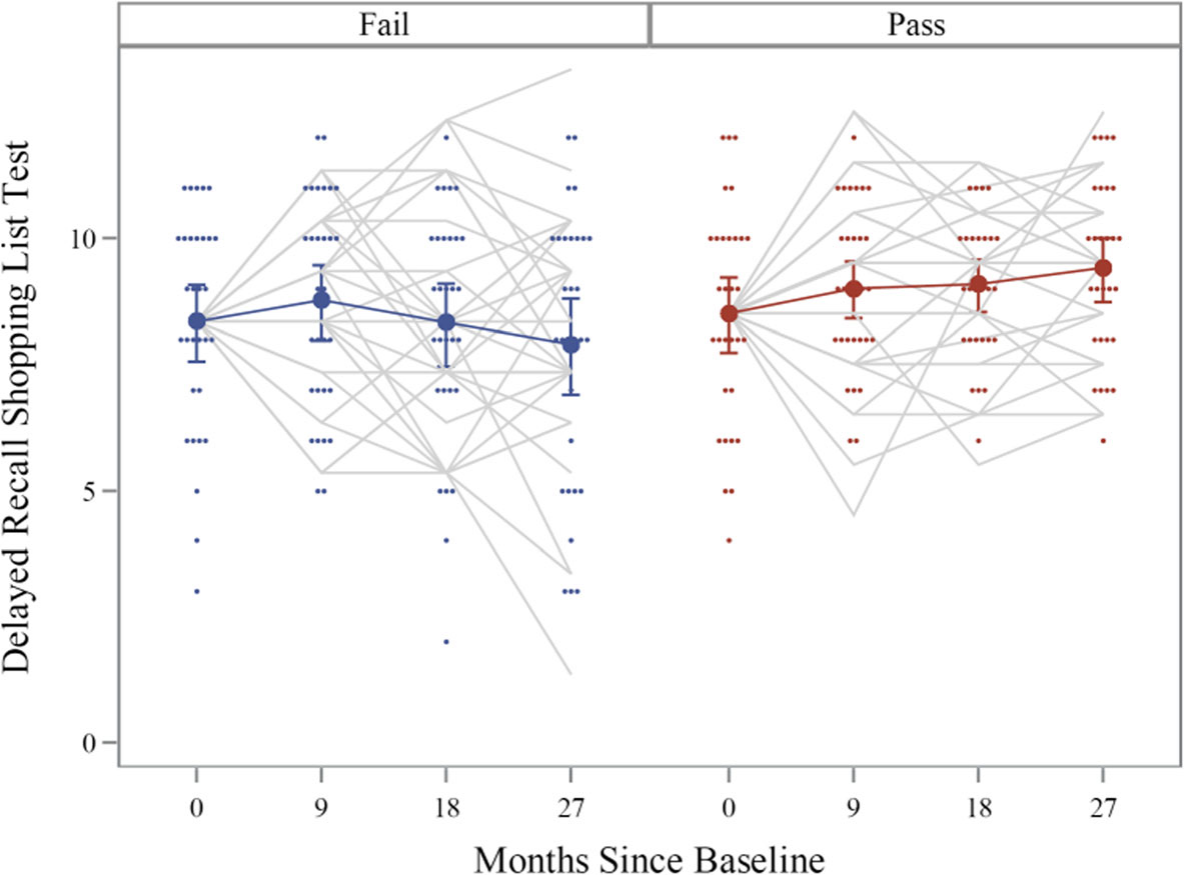
Performance on the International Shopping List (ISLT) Delayed Recall Task, by participants who either failed the scopolamine challenge test (SCT) at baseline (*N* = 28, at end of study) vs. those who passed the SCT at baseline (*N* = 30, at end of study), modeled over all four study visits. Dark lines indicate group mean scores at each visit, with SE bars provided. Both between- and within-subject variation is represented in each group, by displaying individual subject change over time, with each case yoked to the group baseline mean score

Visual inspection of the performance of each group over the course of all four study exams (i.e., at baseline, 9, 18, and 27 months post-baseline) suggested that the group that passed the SCT at baseline benefited from modest practice effects on the ISLT delayed recall task, over the course of the study, whereas those who failed the SCT at baseline did not show evidence of benefiting from continued practice on the test over the study period (see Fig. 1). The GEE model (described above) confirmed this observation (see Table 2) for the ISLT delayed recall.

**Table 2:** Differences in ISLT and GMLT performance between subjects who failed (*N* = 28) vs. passed (*N* = 30) the SCT at baseline, modeled by generalized estimating equation (GEE) over the baseline, 9-, 18-, and 27-month examinations

Task	*p* values for comparison of linear trends across adjusted *p* values and four (4) time points	Method of adjustment
ISLT—Immediate Recall	0.0397*	0.0652 (binomial)
ISLT—Delayed Recall	0.0066*	0.0132* (binomial)
GMLT—Moves/Second	**0.0328**	0.0656 (log normal)
GMLT—Total Errors	**0.0254**	0.0507 (log normal)
GMLT—Delayed Recall		
Total errors	0.2469	0.2469 (log normal)

*p < 0.05

*ISLT* International Shopping List Test, *GMLT* Groton Maze Learning Test

Although the GEE analysis revealed a group difference in performance on the GMLT over the four study visits, it was only the ISLT delayed recall measure that remained significant after adjusting for non-normal distributions (Table 2).

### Decline on ISLT between SCT fail/Aβ+ vs. SCT fail/Aβ− group

**Fig. 2:**
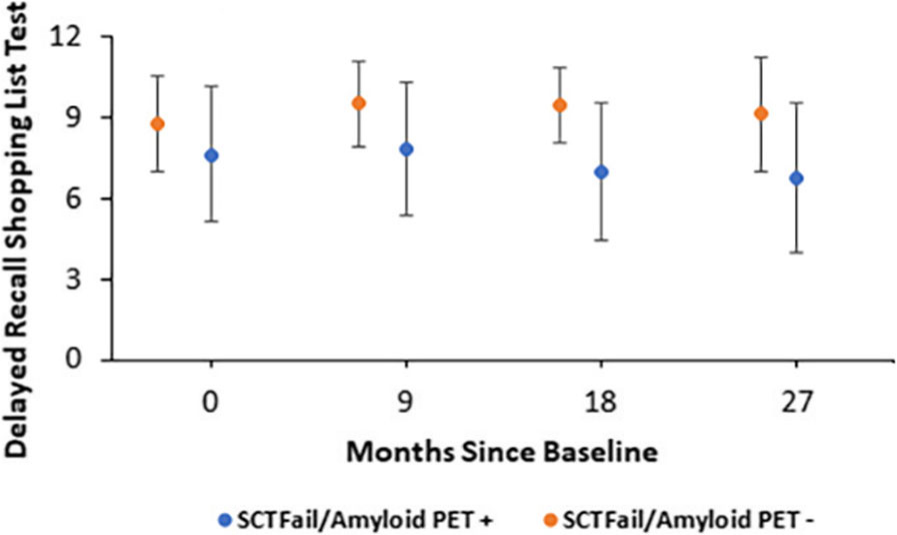
Profile plot showing a significantly reduced performance on the International Shopping List Test (ISLT) Delayed Recall Task for the SCT fail/Aβ+ group compared to the SCT fail/Aβ− group over a 27-month follow-up period. Error bars represent standard deviation from the mean

There was significantly worse ISLT delayed recall task performance at all time points for the SCT fail/Aβ PET+ group compared to the SCT fail/Aβ PET− group (df = 1, MS = 105.67, F = 9.02, *p* = 0.006), with a large effect size (Cohen’s *d* = 1.12) (See Fig. 2).

### ROC analysis results of SCT scores for amyloid PET status at baseline

The proportion of the SCT fail group (0.45) who were Aβ+ was significantly greater than that of the SCT pass group (0.03), *X*(1) = 13.11, *p* = .000294. The area under the curve (AUC) of SCT scores for amyloid PET status is 0.867 (CI = 0.775–0.959). Taking the highest Youden index, an SCT score less than or equal − 0.068 indicated the participant was likely amyloid PET positive with a sensitivity of 1.00 and a specificity of 0.673. Thus, the SCT scores were highly sensitive for amyloid PET status at baseline.

## Discussion

In a group of cognitively normal, healthy, mid-life adults with subjective memory complaints and first-degree family history of AD who all were neurologically within normal limits on a battery of cognitive tests at baseline, we showed that an initial micro-dose scopolamine challenge test (SCT) may both reveal masked prodromal cholinergic defects [11], as well as identify those who show increased neocortical amyloid aggregation and continued cognitive changes over a 27-month follow-up period.

The results of this study support the hypothesis that baseline performance the SCT is related to cognitive changes 27 months later, in association with preclinical AD. The results also show that baseline performance on the SCT is related to PET amyloid imaging results at 27 months, in preclinical AD. Moreover, those who failed the SCT at baseline had greater Aβ accumulation in the ACC over a 27-month period than those who passed the SCT at baseline, indicating that the SCT could be sensitive to amyloid accumulation over time in the earliest stages of the AD pathophysiologic cascade. At the terminus of the study, participants who failed the SCT at baseline also performed significantly worse on an episodic memory test.

Taken together, these results mirror larger cohort studies indicating that Aβ+ preclinical AD patients show a greater longitudinal rate of decline in episodic memory than Aβ− CN older adults [30–33], lending credence to the validity of the SCT as a viable screening tool for preclinical AD. The fact that SCT-related longitudinal cognitive changes were more pronounced in CN, Aβ+ participants could indicate that amyloidosis and cholinergic neurotransmission are either directly or indirectly related in the earliest AD disease stages, and that this relationship persists over at least 27 months.

The absence of cognitive decline in cognitively normal older adults over time in this study is expected and has been shown in larger cohort studies (cf. [33]). However, participants who passed the SCT at baseline showed reliable practice effects on the ISLT over the 27-month period, whereas those who failed the SCT at baseline did not demonstrate expected practice effects. That is, those who failed the SCT generally did not show reliable improvements in cognitive performance as a result of benefitting from repeated exposures to the cognitive test. The absence of this effect within the SCT− group corresponds to literature demonstrating a lack of practice effects in episodic memory tasks in those with preclinical AD [34, 35]. Our data support the hypothesis that lack of practice effects on an episodic memory test could indicate a preclinical AD state in cognitively normal older adults.

Looking more closely at the SCT fail group, participants who were Aβ+ at baseline performed consistently worse than those who failed the SCT and were Aβ− at all time points on an episodic memory test (see Fig. 2), supporting other studies that have shown a reduced episodic memory performance in Aβ+ vs. Aβ− over time [32].

Notably, there were a few individuals in this study who failed the SCT at baseline, but remained Aβ− after a 27-month follow-up period. Importantly, our analyses found that regardless of Aβ PET status, participants who failed the SCT experienced increased cognitive decline over the same follow-up period. These individuals could be experiencing cholinergic disturbance due to non-AD related pathology. Alternatively, cholinergic disturbance is one of the earliest symptoms in the AD pathophysiologic cascade, and these individuals could be particularly susceptible to cholinergic disturbance due to epigenetic, demographic, and other relevant risk factors [36–39]. We suspect, with longer follow-up, these individuals may convert to Aβ+, although further research is required to define the pathophysiologic relationship between amyloid and cholinergic dysfunction in the earliest stages of AD.

As a screening approach, the SCT also has the potential to improve current statistics on screening failure rate for AD prevention trials through increased efficiency. Our ROC analysis at baseline showed that the SCT was a highly sensitive predictor of Aβ PET positivity. A specificity value of 0.673 indicates that the SCT alone may not be able to rule out amyloid pathology in cognitively normal older adults. However, future analyses could examine the specificity of SCT in combination with other factors related to Aβ pathology in preclinical AD, such as episodic memory, family history, and APOE genotype, for detection of Aβ pathology. Presently, screening failure rate in Alzheimer’s prevention clinical trials is estimated at up to 75% [40] and the use of amyloid PET scans as a risk assessment is invasive, and often cost prohibitive. The SCT, due to its relative simplicity and limited resource requirements, has the potential to be used by point-of-care clinicians when PET imaging is not widely available. This will be critical once preventative therapies for AD become available. Using SCT, those at risk who can potentially benefit from longitudinal administration of preventative therapies could potentially be quickly and efficiently identified.

This study has several limitations for consideration. First, the follow-up period was only 27 months, which limits the capacity to track the trajectory of cognitive decline over time, especially in a cognitively normal population. Second, our sample size was small, specifically the sample size of Aβ+ CN participants (*n* = 15), and neocortical amyloidosis does not change dramatically over 27 months, which limits the range of SUVr change over time in our analyses. Also, SCT was only done at baseline and not at the 27-month follow-up visit, which limits our extrapolation of the sensitivity and specificity of SCT scores for Aβ PET status to our baseline data. Future work is required to examine the sensitivity/specificity of the SCT for future conversion to positive Aβ PET in cognitively normal older adults. Despite this limitation, our data show that CN participants who failed the SCT had increased amyloidosis at baseline that persisted at least 27 months. Third, our sample was primarily Caucasian and highly educated, with no comorbid neurological or psychiatric disorders, which may not be representative of the general population. In the future, we plan to replicate this study with a larger, more representative sample and use a longer follow-up period to examine longitudinal cognitive changes. Moreover, our sample was relatively young. In order to validate the SCT as a screening tool, further data collection in a cohort of older adults in the age range where risk for AD is substantially increased is required. Another possible limitation is the length of time required to administer the SCT (5 h). Although this adds to the length of the screening procedure, Aβ PET takes 3–3.5 h to complete, and centers without the staff, equipment, or resources to use this test could eventually use the SCT, which is minimally invasive and considerably more cost efficient. Additionally, future research should examine the use of the SCT as a screening measure in regions where Aβ PET imaging is not readily available. Finally, testing this screening measure in the autosomal dominant AD population presents a unique opportunity to examine the pathophysiological interactions between cholinergic neurotransmission and amyloidosis in the earliest stages of the disease, and could provide valuable insight into the early mechanistic relationships between these two processes that are relevant for the development of preventative therapies.

## Conclusions

SCT is a reliable indicator of continued risk for AD progression and may provide a low-cost, minimally invasive screening method to identify persons at high risk for cortical amyloid aggregation and cognitive decline. Subjects who failed the SCT continued to show significantly greater amyloid binding in the AC after 27 months compared to subjects who passed the SCT (*p* = 0.0046), along with increased rate of amyloid accumulation over the 27-month period as shown on Aβ PET. Besides, those who failed the SCT performed significantly worse on an episodic memory cognitive test (ISLT delayed recall test) than those who passed the SCT at baseline (*p* = 0.025). Only those who passed SCT show evidence of benefiting from continued practice on the test over the study period. Future research should examine the use of the SCT as a screening measure in regions where Aβ PET imaging is not readily available. Exploration of the SCT as a screening measure in an autosomal dominant AD population may allow a unique opportunity to examine the pathophysiological interactions between cholinergic neurotransmission and amyloidosis in the earliest stages of the disease.

## Data Availability

The datasets used and/or analyzed during the current study are available from the corresponding author on reasonable request.

## Abbreviations

Aβ: Beta-amyloid
Aβ−: Amyloid PET negative
Aβ+: Amyloid PET positive
AC: Anterior cingulate
ANCOVA: Analysis of covariance
ANOVA: Analysis of variance
AD: Alzheimer’s disease
APOE: Apolipoprotein
CN: Cognitively normal
DASS: Depression, Anxiety and Stress Scale
GDS: Geriatric Depression Scale
GEE: Generalized estimating equation
GMLT: Groton Maze Learning Test
ISLT: International Shopping List Test
MAC-Q: Memory Complaint Questionnaire
MCI: Mild cognitive impairment
MMSE: Mini-Mental Status Examination
MPS: Moves per second
OBK: One Back Task
OCL: One Card Learning Task
PET: Positron emission tomography
RER: Rule break errors
SCT: Scopolamine challenge test
SUV: Standardized uptake value
SUVr: Standardized uptake value ratio
TER: Total errors
